# Modifiable risk factors of lung cancer in “never-smoker” women

**DOI:** 10.4178/epih/e2015047

**Published:** 2015-10-29

**Authors:** Jong-Myon Bae

**Affiliations:** Department of Preventive Medicine, Jeju National University School of Medicine, Jeju, Korea

**Keywords:** Lung neoplasms, Risk factors, Hormone replacement therapy, Human papillomavirus, Meta-analysis

## Abstract

Korean women with a history of never smoking and with adenocarcinoma showed an increasing trend in lung cancer occurrence during 2002 to 2012. The two modifiable factors of never-smoker lung cancer in women are hormone and oncogenic virus infection. Based on previous studies, hormone replacement therapy (HRT) and human papillomavirus (HPV) infection might afford protection or be a risk factor, respectively. It is necessary to perform a pooled analysis of cohort studies to evaluate HRT and never-smoker lung cancer in women and a systematic review of case-control studies to determine the association between HPV infection and never-smoker lung cancer.

## INTRODUCTION

**Figure 1.**
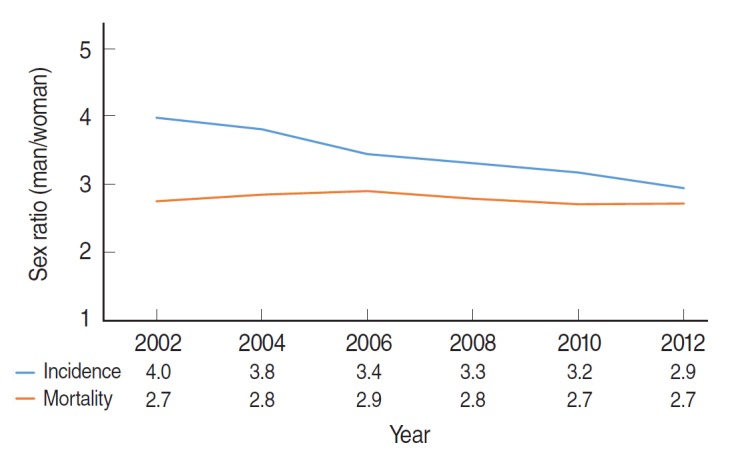
Trends of sex ratios in incidence and mortality rates of lung cancers in Koreans during 2002 and 2012. Source from Korean Statistical Information Service (www.kosis.kr); Korea Central Cancer Registry (www.ncc.re.kr).

**Figure 2.**
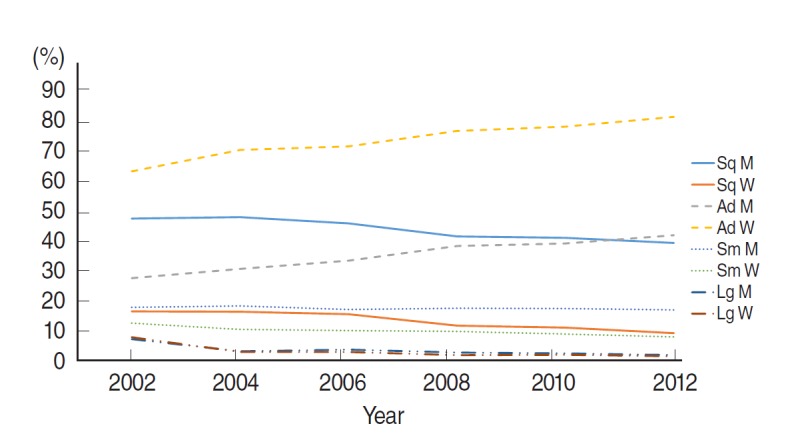
Trands of the histological proportion (%) of lung cancers in Korean during 2002 and 2012. Source from Korea Central Cancer Registry (www.ncc.re.kr). M, men; W, women; Sq, squamous cell carcinoma; Ad, adenocarcinoma; Sm, small cell carcinoma; Lg, large cell carcinoma.

Lung cancer is a primary site cancer that causes the highest number of cancer deaths in Koreans [1]. Smoking is considered the main cause of lung cancer [2,3]. According to cancer incidence and death statistics provided by the Korean Statistical Information Service (www.kosis.kr) and the Korea Central Cancer Registry (KCCR, www.ncc.re.kr), cancer incidence sex ratio decreased steadily for men in comparison to women from 2002 to 2012; there has been no change in mortality rate (Figure 1). In addition, according to the histological distribution of lung cancer patients provided by the KCCR (Figure 2), the incidence of squamous cell carcinoma, which was found primarily in men in 2002, continuously decreased, whereas the incidence of adenocarcinoma increased continuously, becoming the cancer with the highest incidence in 2012. In women, incidence of adenocarcinoma, which accounted for the highest cancer incidence in 2002, increased continuously and accounted for 81% of the lung cancer occurrences in women in 2012. It was consistently reported that 73.0% of women with lung cancer treated in a regional cancer center was never-smoker, and frequency of adenocarcinoma occurrence was higher in never-smokers [4].

Figures 1 and 2 and other articles indicate that occurrence of adenocarcinoma, which has a relatively favorable prognosis among lung cancers, increased in never-smoker women during the past 10 years. As such, since epidemiological characteristics of patients who had never smoked but who had lung cancer (never-smoker lung cancer; NSLCa) were female gender, Asian race, and adenocarcinoma [5,6], NSLCa is determined to be a new disease [7].

## RISK FACTORS OF NEVER-SMOKER LUNG CANCER

Known risk factors of NSLCa include environmental exposures such as second-hand smoke, radon, asbestos, and cooking fumes; genetic susceptibility; hormones; and oncogenic virus [5,6,8]. Of these, the factors closely related to women can be narrowed down to (1) second-hand smoke exposure, (2) cooking fumes, (3) hormones, and (4) infection by human papillomavirus (HPV).

Since cooking fumes or second-hand smoke exposure in childhood has already occurred, they are not modifiable; hence, the effect of a preventive project is limited. In addition, based on genomic analysis of NSLCa, Krishnan et al. [9] claimed that second-hand smoke exposure was unlikely to cause lung cancer in Asians without a smoking history. However, it is clearly necessary to take preventive measures against exposure to second-hand smoke in daily surroundings including the workplace [10]. However, it is not easy to conduct an epidemiological study to determine a cause and effect relationship due to methodological limitations in measuring levels of exposure to second-hand smoke [11,12].

As for female hormones, Siegfried [13] summarized experimental, epidemiological, and clinical evidences that suspected oral contraceptives (OC) and hormone replacement therapy (HRT), and some systematic reviews (SRs) have been performed on these factors. Wu et al. [14] performed SR on results of 9 case-control studies and 5 cohort studies in order to determine the relationship between OC and lung cancer; no significant association was found despite subgroup analysis by two study designs. Three SRs investigated the association between HRT and lung cancer [15-17]. All of them showed statistically significant protective effects of HRT based on meta-analysis of the results of case-control studies. In contrast, meta-analysis results on cohort study data showed no consistency or statistical significance. Thus, it is necessary to conduct an additional cohort study to reveal any relationship between the occurrence of female lung cancer and HRT [18] as well as pooled analysis with already-reported follow-up data of cohort studies.

After the claim by Syrjänen [19] in 1979 that infection by HPV among oncogenic viruses was related with lung cancer occurrence, the prevalence of HPV DNA in lung cancer tissues was studied. SR of the results of such studies [20-23] have reported that about 20% of lung cancer cases were HPV positive, of which the 16 and 18 types were found to be mostly involved. In addition, it has been reported that summary odds ratio by HPV infection was 5.67 times (95% CI, 3.09 to 10.40) more likely based on a SR of case-control studies examining a causal association [24]. As it was found to be associated with lung cancer in never-smoker women [25], HPV vaccination could prevent not only cervical and breast cancer [26], but also lung cancer. However, since it was reported that HPV 16/18 DNA was detected in the blood of lung cancer patients [27], it would be possible to use detection of HPV DNA as a method for early diagnosis of lung cancer in never-smoker women [28].

## SUGGESTION

Of the risk factors for lung cancer in never-smoker women, the grounds of association between HRT history and HPV infection histories, which are modifiable factors, have been reviewed in this study. It is necessary to conduct pooled analysis on cohort studies for HRT history and a SR on case-control studies for HPV infection history. If the findings from these studies show that HRT history is a protective factor against lung cancer incidence and HPV infection history is a risk factor, it would be possible to prepare preventive measures that are more effective.
