# Supplementary material for: Modifiable risk factors of lung cancer in “never-smoker” women
Source: Epidemiol Health. 2015 Oct 29;37:e2015047. doi: 10.4178/epih/e2015047 (PMC4722226; doi:10.4178/epih/e2015047)
Supplement: Supplementary file 1 [file epih-37-e2015047-supplementary.pdf]

<Hypothesis>

비흡연 여성의 폐암 발생과 관련한 교정가능 위험요인

Modifiable Risk Factors of Lung cancer in Never-Smoker Women

국문요약

2002년에서 2012년간 폐암 통계를 살펴보면 비흡연 여성에서 선암종의 발생이 크게 증가하는 것으로 확인된다. 이렇게 여성에서 비흡연 폐암발생과 관련한 요인들로는 호르몬 대체요법 (HRT)과 인유두종바이러스 (HPV) 감염이 알려져 있다. 환자-대조군연구를 이용한 메타분석에 따르면 호르몬대체요법은 폐암발생을 억제하는 것으로 나왔다. HPV에 대한 메타분석은 아직 없는 가운데 위험요인으로 의심하고 있다. 따라서 HRT에 대하여는 코호트 연구 결과를 이용한 연합분석 (pooled analysis)이 필요하며, HPV에 대하여는 환자-대조군연구 결과를 이용한 메타분석이 수행될 필요가 있다.

중심어: 폐암, 위험요인, 호르몬대체요법, 인유두종바이러스, 메타분석

폐암은 한국인 암 사망의 수위를 차지하는 원발암 (primary site cancer)으로 [1], 흡연이 폐암의 주된 발생 요인으로 보고 있다 [2,3]. 국가통계포털 (www.kosis.kr; KOSIS)과 한국중앙암등록본부 (www.ncc.re.kr; KCCR)가 제공한 암 발생 및 사망통계에 따르면, 2002년부터 2012년간 암발생률과 사망률에 있어 여자를 기준으로 남자의 성비 (sex ratio)가 사망률에서는 변동이 없는 반면, 발생률에서는 꾸준히 감소하였다 (Fig. 1). 또한 KCCR이 제공한 폐암 발생자의 조직학적 분포를 살펴보면 (Fig. 2), 2002년도 기준으로 남자에서 가장 많았던 편평세포암 (squamous cell carcinoma)은 꾸준히 감소하는 반면, 선암 (adenocarcinoma)은 꾸준히 증가하여 2012년 1위가 되었다. 여자에는 2002년 기준으로 가장 많았던 선암은 그 이후 계속 증가하여 2012년 현재 여성 폐암발생 중 81%를 차지하였다. 이와 더불어 한 지역 암센터에서 치료한 여성 폐암 대부분은 비흡연자들 (73.0%)이며, 비흡연자에서 선암의 빈도가 높다고 보고하였다 [4].

이상의 두 그래프와 발표의 의미는 지난 10년간 비흡연 여성에서 예후가 상대적으로 좋은 선암의 폐암 발생이 많아졌다는 것으로 요약할 수 있다. 이처럼 평생을 통해 흡연한 적이 없지만 폐암이 생기는 경우 (Never-smokers lung cancer, NSLCa)에 있어, 여성, 아시아인, 선종이 더 많다는 점 등 [5.6]의 역학적 특성을 보이면서 새로운 질병으로 인식해야 한다는 주장이 제기되고 있다 [7].

NSLCa 위험요인으로 알려진 것들로는 간접흡연 (second-hand smoking), 라돈 (radon), 석면 (asbestos), 조리시 연기 (cooking fumes) 등의 환경폭로, 유전적 감수성 (genetic susceptability), 호르몬, 암유발 바이러스 (oncogenic virus) 등이 있다 [5,6,8]. 이들 중 여성과 밀접한 관련이 있다고 보는 것으로 (1) 간접흡연 (2) 조리시 연기 (3) 호르몬 (4) 인유두종바이러스 (human papillomavirus, HPV) 감염으로 좁힐 수 있다.

이중 조리시 연기나 어릴 때 간접흡연은 이미 노출이 되어서 개입이 불가능 (modifiable) 하다는 점에서 예방사업의 효과는 제한적이다. 이에 더하여, Krishnan et al. [9]은 NSLCa의 유전체 분석을 근거로 흡연력이 없는 아시아인의 폐암발생에 있어 간접흡연 가능성은 낮다는 주장을 했다. 그렇지만 일상생활 속에서 직장 동료 등 주변으로 부터의 간접흡연 노출에 대한 예방적 조치가 필요한 것은 분명하다 [10]. 문제는 간접흡연의 노출정도를 측정하는 방법론적 한계 때문에 인과규명을 위한 역학연구 수행이 쉽지 않다는 점이다 [11,12].

여성 호르몬에 있어서는 경구피임제 (oral contraceptives, OC)와 호르몬대체요법 (hormone replacement therapy, HRT)를 의심하는 실험실적, 역학적, 임상적 근거들을 Syegfried 가 정리하였으며 [13], 이에 대한 메타분석들이 수행되어 왔다. Wu et al. [14]은 OC와 폐암간의 관련성을 알아보기 위하여 9편의 환자-대조군연구와 5편의 코호트 연구 결

과를 선정하여 체계적 고찰을 시행한 결과 관련성이 없는 것으로 나왔다. 두 가지 연구 설계별로 하부군 분석 (subgroup analysis)을 했지만 여전히 의미를 찾을 수 없었다. 그리고 HRT 와 폐암간의 관련성을 알아본 체계적 고찰은 3편이 있다 [15-17]. 이들 모두 환자-대조군 연구 결과의 메타분석은 보호 효과가 있으며 통계적으로 유의하게 나왔다. 그러나 코호트 연구에 대한 메타분석은 일관성이 없으면서 통계적 유의성을 보이지 않았다. 따라서 HRT에 대한 여성 폐암발생을 규명하는 코호트 연구가 추가로 필요할 뿐만 아니라 [18], 기존의 발표된 코호트 추적자료들을 모아 연합분석 (pooled analysis)을 시행할 필요가 있다.

암유발바이러스 중 HPV 감염이 폐암 발생과 관련이 있다고 1979년 Syrjänen [19]이 주장한 이후, 폐암 조직에서 HPV DNA 유병률 (prevalence)에 관한 연구들이 수행되었다. 이들에 대한 체계적 고찰 [20-23] 결과, 발생한 폐암의 약 20%에서 HPV 양성이며, 16과 18 유형이 가장 많이 관여하는 것으로 보고되었다. 또한 인과규명을 위한 환자-대조군 연구의 체계적 고찰에서 HPV 감염으로 인한 SOR이 5.67배 (95% CI: 3.09-10.40)로 보고하였다 [24]. 비흡연 여성에 있어서도 여전히 관련성이 있다고 나온다는 점에서 [25], HPV 예방접종은 자궁경부암, 유방암 [26] 뿐만 아니라, 폐암 발생까지 예방할 가능성이 열리게 된다. 한편, 폐암환자의 혈액에서도 HPV 16/18 DNA가 검출된다는 보고 [27]에 따르면, 비흡연 여성에 있어 폐암의 조기 진단으로도 활용할 가능성이 있겠다 [28].

지금까지 비흡연 여성에서의 폐암 발생에 관여하는 위험 요인들 중 특히 교정 가능한 (modifiable) 요인들인 HRT 복용력과 HPV 감염력에 있어 그 관련성 근거들을 살펴보았다. 향후의 과제들로는 HRT 복용력에 대하여 코호트 연구에 대한 연합분석이 필요하며, HPV 감염력에 대하여는 환자-대조군 연구에 대한 체계적 고찰이 필요하다. 이를 통해 만약

HRT 복용력이 폐암 발생의 보호요인이고, HPV 감염력이 위험요인이라면, 이를 반영한 보다 적극적인 예방대책을 수립할 수 있을 것이다.

감사의 글

폐암 등록환자의 2002-2012년도 조직학적 분류에 관한 정보 제공에 협조해준 한국중앙암등록본부에 감사를 표합니다.

References

1. Jung KW, Won YJ, Kong HJ, Oh CM, Cho H, Lee DH, et al. Cancer statistics in Korea: incidence, mortality, survival, and prevalence in 2012. Cancer Res Treat 2015;4:127-141.

2. Bae JM, Li ZM, Shin MH, Kim DH, Lee MS, Ahn YO. Lung cancer incidence by smoking status in Korean men: 16-years of observations in the Seoul Male Cancer Cohort Study. J Korean Med Sci 2013;28:636-637.

3. Dela Cruz CS, Tanoue LT, Matthay RA. Lung cancer: epidemiology, etiology, and prevention. Clin Chest Med 2011;32:605-644.

4. Kang HW, Park CW, Kim WJ, Song SY, Na KJ, Jeong JU, et al. Never-smoker lung cancer is increasing. J Lung Cancer 2012;11:89-93.(Korean)

5. Subramanian J, Govindan R. Lung cancer in 'Never-smokers': a unique entity. Oncology 2010;24:29-35.

6. Alberg AJ, Wallace K, Silvestri GA, Brock MV. Invited commentary: the etiology of lung cancer in men compared with women. Am J Epidemiol 2013;177:613-616.

7. Sun S, Schiller JH, Gazdar AF. Lung cancer in never smokers—a different disease. Nat Rev Cancer 2007;7:778-790.

8. Planchard D, Besse B2. Lung cancer in never-smokers. Eur Respir J 2015;45:1214-1217.

9. Krishnan VG, Ebert PJ, Ting JC, Lim E, Wong SS, Teo AS, et al. Whole-genome sequencing of asian lung cancers: second-hand smoke unlikely to be responsible for higher incidence of lung cancer among Asian never-smokers. Cancer Res 2014;74:6071-6081.

10. Besaratinia A, Pfeifer GP. Second-hand smoke and human lung cancer. Lancet Oncol 2008;9:657-666.

11. Jaakkola MS, Jaakkola JJ. Assessment of exposure to environmental tobacco smoke. Eur Respir J 1997;10:2384-2397.

12. Kawaguchi T, Ando M, Kubo A, Takada M, Atagi S, Okishio K, et al. Long exposure of environmental tobacco smoke associated with activating EGFR mutations in never-smokers with non-small cell lung cancer. Clin Cancer Res 2011;17:39-45.

13. Siegrfied JM. Smoking out reproductive hormone actions in lung cancer. Mol Cancer Res 2014;12(1):24-31.

14. Wu W, Yin ZH, Guan P, Ren YW, Zhou BS. Association of oral contraceptives use and lung cancer risk among women: an updated meta-analysis based on cohort and case-control studies. Asian Pac J Cancer Prev 2014;15:1205-1210.

15. Oh SW, Myung SK, Park JY, Lym YL, Ju W. Hormone therapy and risk of lung cancer: a meta-analysis. J Womens Health 2010;19:279-288.

16. Chen X, Cai L. Meta-analysis of the effects on hormone replacement therapy and oral contraceptives associated with female lung cancer risk. Wei Sheng Yan Jiu 2009;38:672-676. (Chinese)

17. Yao Y, Gu X, Zhu J, Yuan D, Song Y. Hormone replacement therapy in females can decrease the risk of lung cancer: a meta-analysis. PLoS One 2013;8:e71236.

18. Bae JM, Kim EH. Hormonal replacement therapy and risk of lung cancer in women: an adaptive meta-analysis of cohort studies. J Prev Med Public Health 2015. (in press)

19. Syrjänen KJ. Condylomatous changes in neoplastic bronchial epithelium. Report of a case. Respiration 1979;38:299-304.

20. Srinivasan M, Taioli E, Ragin CC. Human papillomavirus type 16 and 18 in primary lung cancers—a meta-analysis. Carcinogenesis 2009;30:1722-1728.

21. Hasegawa Y, Ando M2, Kubo A3, Isa S4, Yamamoto S4, Tsujino K5, et al. Human papilloma virus in non-small cell lung cancer in never smokers: a systematic review of the literature. Lung Cancer 2014;83:8-13.

22. Klein F, Amin Kotb WF, Petersen I. Incidence of human papilloma virus in lung cancer. Lung Cancer 2009;65:13-18.

23. Syrjänen K. Detection of human papillomavirus in lung cancer: systematic review and meta-analysis. Anticancer Res 2012;32:3235-3250.

24. Zhai K, Ding J, Shi HZ. HPV and lung cancer risk: a meta-analysis. J Clin Virol 2015;63:84-90.

25. Bae JM, Kim EH. Human papillomavirus infection and risk of lung cancer in never-smokers and women: an adaptive meta-analysis for case-control studies. Epidemiol Health. 2015. (in press)

26. Bae JM. Two hypotheses of dense breasts and viral infection for explaining incidence of breast cancer by age group in Korean women. Epidemiol Health 2014;36:e2014020.

27. Chiou HL, Wu MF, Liaw YC, Cheng YW, Wong RH, Chen CY, et al. The presence of human papillomavirus type 16/18 DNA in blood circulation may act as a risk marker of lung cancer in Taiwan. Cancer 2003;97:1558-1563.

28. Li YJ, Tsai YC, Chen YC, Christiani DC. Human papilloma virus and female lung adenocarcinoma. Semin Oncol 2009;36:542-552.

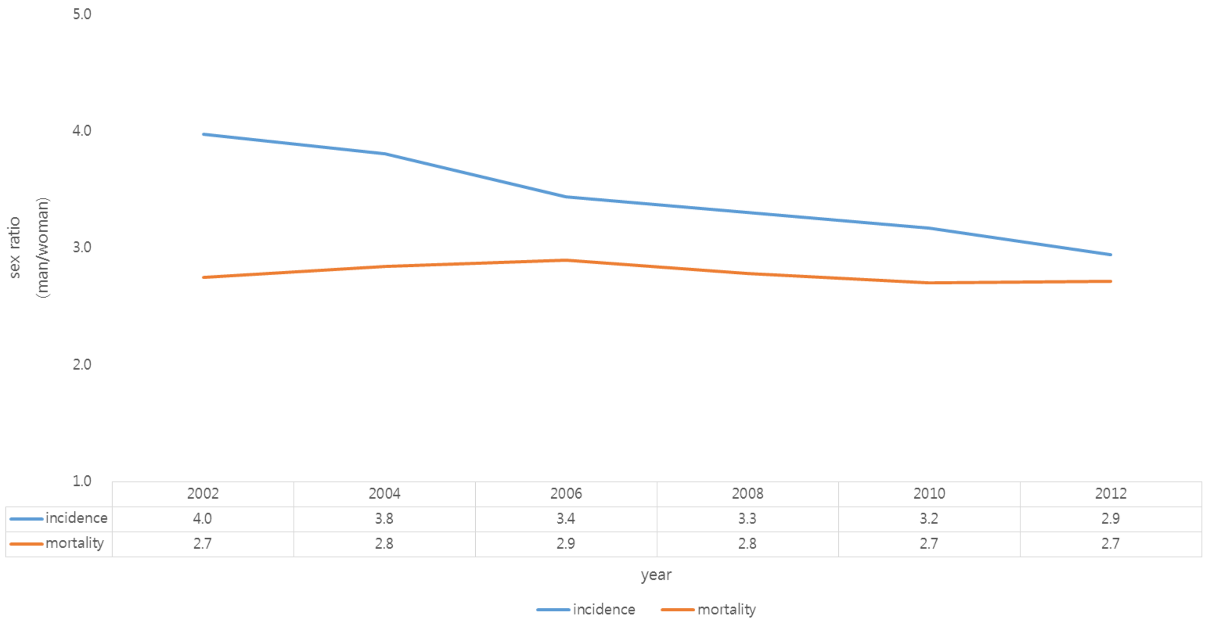

	2002	2004	2006	2008	2010	2012
incidence	4.0	3.8	3.4	3.3	3.2	2.9
mortality	2.7	2.8	2.9	2.8	2.7	2.7

Fig 1. Trends of sex ratios in lung cancer incidence and morality rates in Koreans during 2002 through 2012. Sources: KOSIS [www.kosis.kr] and KCCR [www.ncc.re.kr]

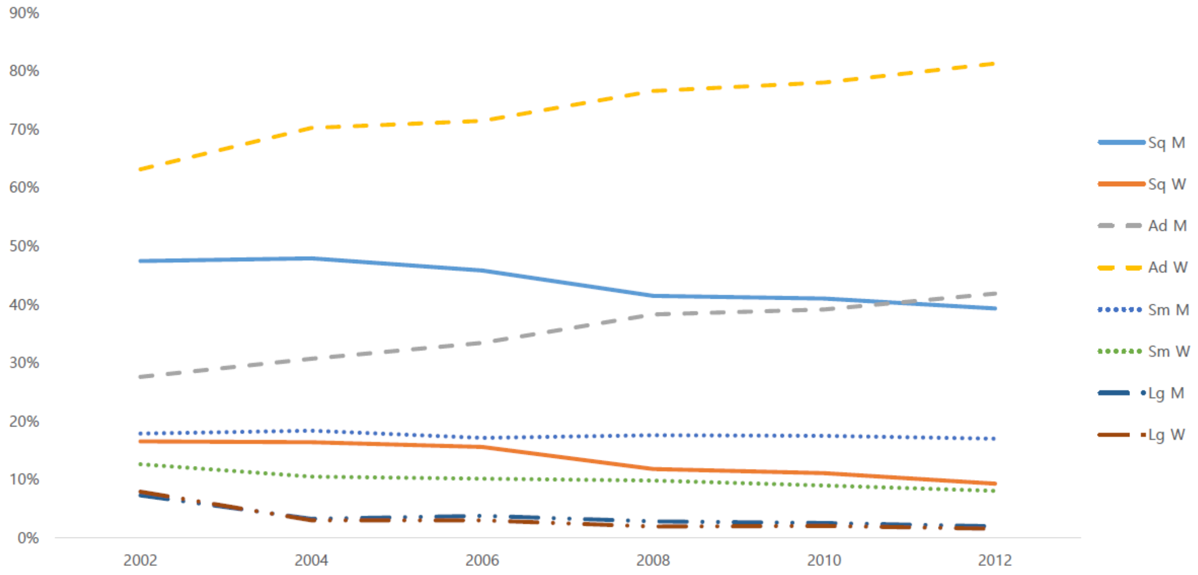

Fig 2. Trends of the histological proportion (%) of lung cancers in Koreans during 2002 through 2012. Sources: KCCR [www.ncc.re.kr]. M (men), W (women) Sq (squamous cell carcinoma), Ad (adenocarcinoma), Sm (small cell carcinoma), Lg (large cell carcinoma).
